# The impact of a remote monitoring system of healthcare resource consumption in patients on automated peritoneal dialysis (APD): A simulation study 

**DOI:** 10.5414/CN109471

**Published:** 2018-08-14

**Authors:** Kiyotaka Uchiyama, Naoki Washida, Nobuyuki Yube, Takahiro Kasai, Keisuke Shinozuka, Kohkichi Morimoto, Akihito Hishikawa, Hiroyuki Inoue, Hidenori Urai, Aika Hagiwara, Kentaro Fujii, Shu Wakino, Souzana Deenitchina, Hiroshi Itoh

**Affiliations:** 1 Division of Endocrinology, Metabolism, and Nephrology, Department of Internal Medicine, Keio University School of Medicine, Tokyo,; 2 Department of Nephrology, International University of Health and Welfare School of Medicine, Chiba, and; 3 Department of Medical Affairs, Baxter Ltd., Tokyo, Japan

**Keywords:** automated peritoneal dialysis, healthcare resource, remote monitoring

## Abstract

Aims: Remote monitoring (RM) can improve management of chronic diseases. We evaluated the impact of RM in automated peritoneal dialysis (APD) in a simulation study. Materials and methods: We simulated 12 patient scenarios with common clinical problems and estimated the likely healthcare resource consumption with and without the availability of RM (RM+ and RM– groups, respectively). Scenarios were evaluated 4 times by randomly allocated nephrologist-nurse teams or nephrologist-alone assessors. Results: The RM+ group was assessed as having significantly lower total healthcare resource consumption compared with the RM– group (36.8 vs. 107.5 total episodes of resource consumption, p = 0.002). The RM+ group showed significantly lower “unplanned hospital visits” (2.3 vs. 11.3, p = 0.005), “emergency room visits” (0.5 vs. 5.3, p = 0.003), “home visits” (0.5 vs. 5.8, p = 0.016), “exchanges over the telephone” (18.5 vs. 57.8, p = 0.002), and “change to hemodialysis” (0.5 vs. 2.5, p = 0.003). Evaluations did not differ between nephrologist-nurse teams vs. nephrologist-alone assessors. Conclusion: RM can be expected to reduce healthcare resource consumption in APD patients.

## Introduction 

In Japan, the number of dialysis patients has exceeded 320,000 [1], leading to increased healthcare expenditure in hemodialysis (HD) clinics. Peritoneal dialysis (PD) can be done at home, enabling treatment to be adapted to a patient’s lifestyle, and it can be provided at a lower cost. Automated peritoneal dialysis (APD) involves the use of a device that performs PD exchanges for the patient while the patient is asleep, which improves the patient’s quality of life (QoL) and experience of dialysis [2, 3]. 

Recently, remote monitoring (RM) has been successfully used to manage diseases such as chronic obstructive pulmonary disease and congestive heart failure [4, 5, 6]. For example, RM can reduce hospitalizations and deaths in heart failure patients by 29% and 20%, respectively [7, 8]. It is therefore plausible that RM has similar clinical benefits for PD patients, with better patient-centered outcomes such as improved QoL. The ability to remotely load a patient’s prescription and observe their response to therapy through monitoring of drainage volumes, vital signs, and other parameters can be expected to reduce complications and adverse events, and thereby reduce healthcare resource consumption [9]. 

In Japan, RM is widely utilized for implantable cardiac devices, and it is associated with marked improvements in efficiencies of care [10, 11]. In contrast, RM for APD devices is not currently available in Japan. Therefore, we assessed the impact of RM on healthcare resource consumption using simulated clinical scenarios [12]. We prepared these simulated clinical scenarios with hypothetical APD patients and compared their likely resource consumption in the presence or absence of RM. These clinical scenarios were then judged by experienced healthcare professionals in an attempt to recreate a clinical RM trial for PD patients. 

## Methods 

### Study design 

This study was approved by the Keio University Hospital Ethics Review Committee (approval number: 20160200) and conducted in accordance with the 1964 Declaration of Helsinki and its later amendments or comparable ethical standards. Written consent was obtained from all participants. 

**Figure 1.**
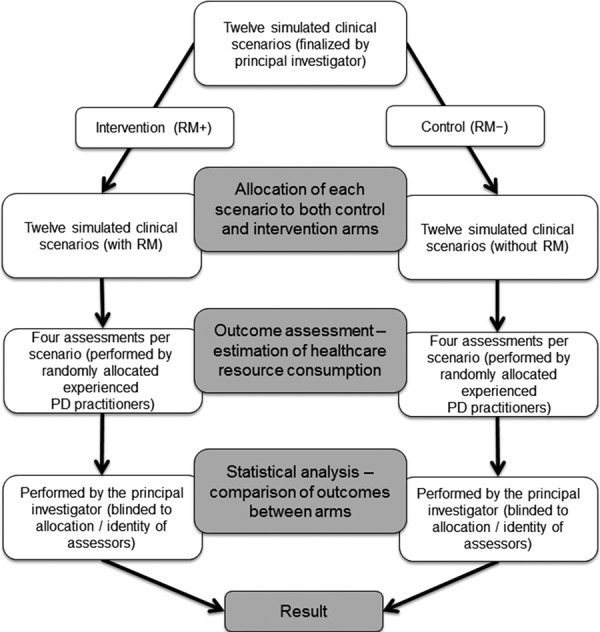
Study design. PD = peritoneal dialysis; RM = remote monitoring.

This was a collaborative study between Keio University Hospital and Baxter Japan Ltd. and was conducted in the Keio University Hospital nephrology network in Tokyo, Japan. The overall study design is presented in Figure 1. Hypothetical patients, each with a different PD-related problem, received either usual clinical care with the availability of RM from the caring team (RM+ group) or usual clinical care without the availability of RM (RM– group). Clinical scenarios were randomly allocated to independent groups of experienced practitioners who then assessed healthcare resource consumption. 

### Simulated patient population 

The simulated clinical scenarios included 12 simulated patients each with unique clinical characteristics. These scenarios were based on examples previously developed by the global medical affairs group at Baxter International Healthcare in 2015 but were modified by the principal investigator of this study to reflect typical Japanese APD patients and practice. Each of the 12 simulated clinical scenarios modeled a different PD-related problem and was presented as a narrative describing the hypothetical patient and their PD-related complication (Supplemental Material). 

### Intervention 

Remote monitoring involved an Internet-based system that collects information from patients, such as their treatment data, blood pressure and body weight, and uploads prescriptions directly from the healthcare professional to the APD machine. 

### Outcome 

The primary outcome of the study was to estimate healthcare resource consumption for each scenario under RM+ and RM– conditions. Overall consumption was quantified in nine different categories: unplanned hospital visits, emergency room visits, home visits, exchanges over the telephone, device swap (including change of the prescription), change to hemodialysis, hospitalizations, retraining, and other (medical audit, additional prescriptions, additional blood tests, domiciliary care, retraining for nurses). 

Outcomes were assessed by experienced healthcare professionals who were randomly allocated to estimate healthcare resource consumption for each clinical scenario. The inclusion criteria defining “experienced” healthcare professionals included: 1) employed and practicing as a credentialed nephrologist or nephrology nurse; 2) cumulative clinical experience of directly managing ≥ 20 APD patients; and 3) ≥ 3 years working in a PD department within the hospital network. 

A total of 8 doctors and 4 nurses were organized into 8 teams: 4 consisting of a nephrologist and a nephrology nurse (“nephrologist-nurse teams”), and 4 consisting of a single nephrologist (“nephrologist-alone”). Each team was allocated to either the RM+ or RM– of a given scenario and tasked to review the scenario in their narrative using “+” or “–” on the survey form to indicate likely healthcare resource consumption (Supplemental Material). 

### Statistical analysis 

For the main analysis, the unpaired Student’s t-test and the Mann-Whitney U-test were used to compare estimated healthcare resource consumption in the intervention (RM+) and control (RM–) arms. In the case of non-normally-distributed data, the Mann-Whitney U-test served as a sensitivity analysis. In the supplementary analysis, estimates were compared between the nephrologist-nurse teams and the nephrologist-alone assessors. All hypothesis testing was done with a significance level of p < 0.05, using R version 3.3.0 for OSX software. The data analyst could not be blinded to the intervention but was blinded to the allocation and identity of outcome assessors. 

Results are presented as mean ± standard deviation and median (interquartile range (IQR). 

## Results 

### Main results 

Total healthcare resource consumption was significantly lower in the RM+ group (36.8 ± 5.4 events) than in the RM– group (107.5 ± 26.7 events) across all scenarios (Student’s t-test p = 0.002) (Table 1, Supplementary Table 1, and Supplementary Figure 1). This equated to a decrease of 70.8 events across all 12 simulated patients (Table 1), and an average reduction of 5.9 events per simulated patient. 

The RM+ group showed significantly lower resource consumption in the following categories: unplanned hospital visits (2.3 ± 1.0 vs. 11.3 ± 4.0 events, p = 0.005); emergency room visits (0.5 ± 0.6 vs. 5.3 ± 1.9 events, p = 0.003); home visits (0.5 ± 1.0 vs. 5.8 ± 3.0 events, p = 0.016); exchanges over the telephone (18.5 ± 2.6 vs. 57.8 ± 14.6, p = 0.002); change to hemodialysis (0.5 ± 0.6 vs. 2.5 ± 0.6, p = 0.003), and other (including medical audit, additional prescription, blood tests,****domiciliary care, retraining for nurses) (3.3 ± 0.5 vs. 5.8 ± 1.0, p = 0.004) (Table 1). 

The greatest reduction was observed in the number of exchanges over the telephone, with a difference between groups of 39.3 events across all 12 simulated patients (Table 1), and an average reduction of 3.3 per simulated patient. 

### Additional results 

**Figure 2.**
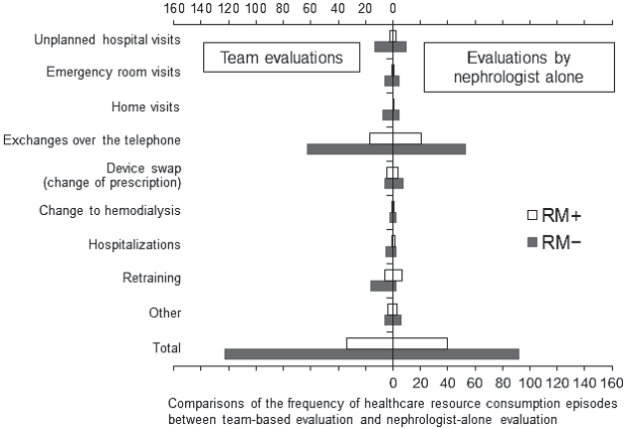
Comparisons of the frequency of healthcare resource consumption episodes between team-based evaluation and nephrologist-alone evaluation. No significant difference was observed between the two groups for any of the resources. RM = remote monitoring.

There was no significant difference between evaluations by the nephrologist-nurse teams vs. the nephrologist-alone assessors (Figure 2). 

## Discussion 

Our study shows that RM is expected to improve efficiencies of care and therefore reduce healthcare resource consumption in APD patients facing common clinical problems. Although our study is a simulation, with expert but still opinion-based outcome assessment, any results are therefore only directional and suggestive. Nonetheless, this study shows the potential improvements in PD outcomes, and that any additional gain in technique survival will translate to increased PD prevalence, and therefore reduce the overall expenditure on end-stage kidney failure care [13]. 

Of the few studies in the literature exploring the use of RM for PD, most report findings similar to ours [14, 15, 16, 17, 18, 19, 20, 21]. For instance, Gallar et al. [15] reported reduced clinical examination time for PD patients using home videoconferencing equipment compared with hospital consultation (22 vs. 33 minutes, p < 0.05), and reduced hospitalization rates (2.2 vs. 5.7 days/patient/year, p < 0.05). Other studies suggest that such improvement in process outcomes might translate to actual clinical benefits. In a retrospective observational study of the RM system in rural and urban PD patients, the 5-year survival was unexpectedly high, especially in rural patients [20, 21]. 

In Japan, Nakamoto et al. [18] were the first to develop a telemedicine system for PD, which has been progressively upgraded as telecommunication devices technology has advanced [17, 19]. Here, telemedicine was particularly advantageous for elderly and disabled patients and was shown to minimize the burden of outpatient visits as well as providing real-time information on those who might be more vulnerable than usual when receiving care at home. Of course, there are important differences between the implementation of telemedicine as reported by Nakamoto et al. [17, 18, 19] and our simulation. The intervention we modeled is a comparatively basic one; however, it still supplied increased support to patients during home-based medical care with greater and timelier availability of information about treatment delivery as well patients’ response to therapy. 

In our study, we examined the possible impact of RM on poor treatment adherence, which is associated with significant increases in mortality, technique failure, and hospitalization in PD patients [22]. Of note, our results showed that RM can be expected to reduce the frequency of healthcare resource consumption even in nonadherent patients (data not shown). The ability of RM to easily and quickly check treatment prescriptions as well as infusion/drainage volumes, vital signs, and other parameters is an important advance for these particularly difficult patients. 

RM is cost-effective for managing chronic obstructive pulmonary disease and congestive heart failure, with emerging positive economic data in diabetes care, management of elevated cardiovascular risk, and perhaps even depression [23, 24, 25, 26, 27, 28, 29]. Cost-savings in these scenarios are driven by improved efficiencies in care and reduced complications. For PD, data from a recent economic evaluation showed that most elderly APD patients were hospitalized at least once during the first year of therapy, averaging ~ 20 days in hospital at a cost of ~ 5,000 USD of medical fees per hospitalization (https://mfeesw.net/tr/dpc) [13, 30]. Reducing complications in PD will ensure sustainable and accessible healthcare in the increasingly aged and comorbid Japanese population. 

Our study used a simulation approach, which is an established way to model health resource utilization [31, 32, 33, 34, 35, 36, 37]. However, there are three major limitations to the simulation approach. First, health economic and outcome studies that use discrete-choice experiments and Markov models are standard, but are critically dependent on whether the simulated model is both reasonably accurate and persuasive. This depends on high-quality data and cumulative clinical experience informing the simulation. However, real clinical situations are often more complex, with assumptions and limitations that would impair any resulting analysis if they were fully described. For this reason, simulations are often simplistic and more persuasive than would otherwise be desirable. In our study, the scenarios were pressure-tested among senior nephrologists at Baxter International Healthcare and the Keio University Hospital network. The evaluators could adjust their ratings of resource consumption up or down. If they did not believe the scenario to be plausible, they could readily rate the consumption to be higher, rather than lower, with RM. 

Of note, the Keio University Hospital network is already implementing a remote monitoring system for primary-care patients (Primary care Keio Community study (PKC study), Ethics Review Committee approval 2014-373). As a result, we have some preliminary experience regarding the impact of RM on the clinical care of home-dialysis patients. Our simulations are therefore based on both published literature and our clinical experience. Although our methodology is standard for simulation studies in health economic literature and compares well with other studies, our results should only ever be considered as directional rather than definitive. 

Another limitation of our study is that it involved opinion-based outcome assessments, which are inherently subjective. Finally, our study has limited generalizability due to its single-center sampling frame, and the possibility that local treatment policies in the Keio University Hospital may have directly influenced estimates by outcome assessors, who were all sourced from this hospital network. 

Our study is a scoping exercise, and further studies are needed to definitively study RM in APD patients and evaluate the impact on clinical and patient-centered outcomes as well as healthcare resource consumption in a real-world setting. The results of the present simulation study suggest that the use of RM contributes not only to reduced healthcare resource consumption, but also to improved clinical outcomes. Further studies are needed to confirm the present findings in real APD patients using APD devices with RM function. 

## Acknowledgment 

The authors thank Baxter USA for providing the draft scenarios, and the nurses from the Department of Nephrology, Endocrinology, and Metabolism, Keio University Hospital, for evaluating the scenarios. The authors also thank Associate Professor Mark R. Marshall for his advice in formatting and writing this manuscript. 

## Funding 

This study was conducted with the financial support of collaborating partner, Baxter Ltd. 

## Conflict of interest 

This study was conducted with the financial support of the collaborating partner, Baxter Ltd. NY and SD are employees of Baxter Ltd. 

**Table 1.** Frequency of healthcare resource consumption episodes in the RM+ and RM– groups, summarized across the four assessments of each scenario (Student’s t-test).

Healthcare resource	RM+ (n = 12)	RM– (n = 12)	Mean difference between RM+ and RM–	Unpaired t-test p-value (RM+ vs. RM–)
Unplanned hospital visits	2.3 ± 1.0	11.3 ± 4.0	9.0	0.005
Emergency room visits	0.5 ± 0.6	5.3 ± 1.9	4.8	0.003
Home visits	0.5 ± 1.0	5.8 ± 3.0	5.3	0.016
Exchanges over the telephone	18.5 ± 2.6	57.8 ± 14.6	39.3	0.002
Device swap (change of prescription)	4.0 ± 2.3	6.5 ± 1.3	2.5	0.108
Change to hemodialysis	0.5 ± 0.6	2.5 ± 0.6	2.0	0.003
Hospitalizations	1.3 ± 1.5	3.5 ± 1.9	2.3	0.114
Retraining	6.0 ± 1.4	9.3 ± 12.6	3.3	0.626
Other	3.3 ± 0.5	5.8 ± 1.0	2.5	0.004
Total	36.8 ± 5.4	107.5 ± 26.7	70.8	0.002

RM = remote monitoring. Data are shown as mean ± standard deviation.

## Supplemental material

Supplemental material Supplemental Tables, Figures, and Patient Scenarios.
