# Supplementary material for: The impact of a remote monitoring system of healthcare resource consumption in patients on automated peritoneal dialysis (APD): A simulation study
Source: Clin Nephrol. 2018 Aug 14;90(5):334–40. doi: 10.5414/CN109471 (PMC6206566; doi:10.5414/CN109471)
Supplement: Supplemental material [file clinnephrol-90-334-S01.pdf]

Supplementary Table 1. Comparison of the frequency of healthcare resource consumption episodes between RM+ and RM− evaluations (Mann–Whitney U test)

Healthcare resource	Median (interquartile range)		Mann–Whitney U test *P* value (RM+ vs. RM−)
	RM+ (n=12)	RM− (n=12)	
Unplanned hospital visits	2.5 (1.3–3.0)	10.0 (8.3–15.5)	0.020
Emergency room visits	0.5 (0–1.0)	4.5 (4.0–7.3)	0.019
Home visits	0 (0–1.5)	6.0 (2.8–8.5)	0.026
Exchanges over the telephone	18.0 (16.3–21.3)	58.0 (43.5–71.8)	0.021
Device swap (change of prescription)	4.0 (2.0–6.0)	6.5 (5.3–7.8)	0.137
Change to hemodialysis	0.5 (0–1.0)	2.5 (2.0–3.0)	0.018
Hospitalizations	1.0 (0–2.8)	3.0 (2.0–5.5)	0.137
Retraining	6.5 (4.5–7.0)	4.0 (1.8–22.0)	0.372
Other	3.0 (3.0–3.8)	5.5 (5.0–6.8)	0.017
Total	37.0 (31.5–41.8)	105.5 (83.8–133.3)	0.021

RM, remote monitoring

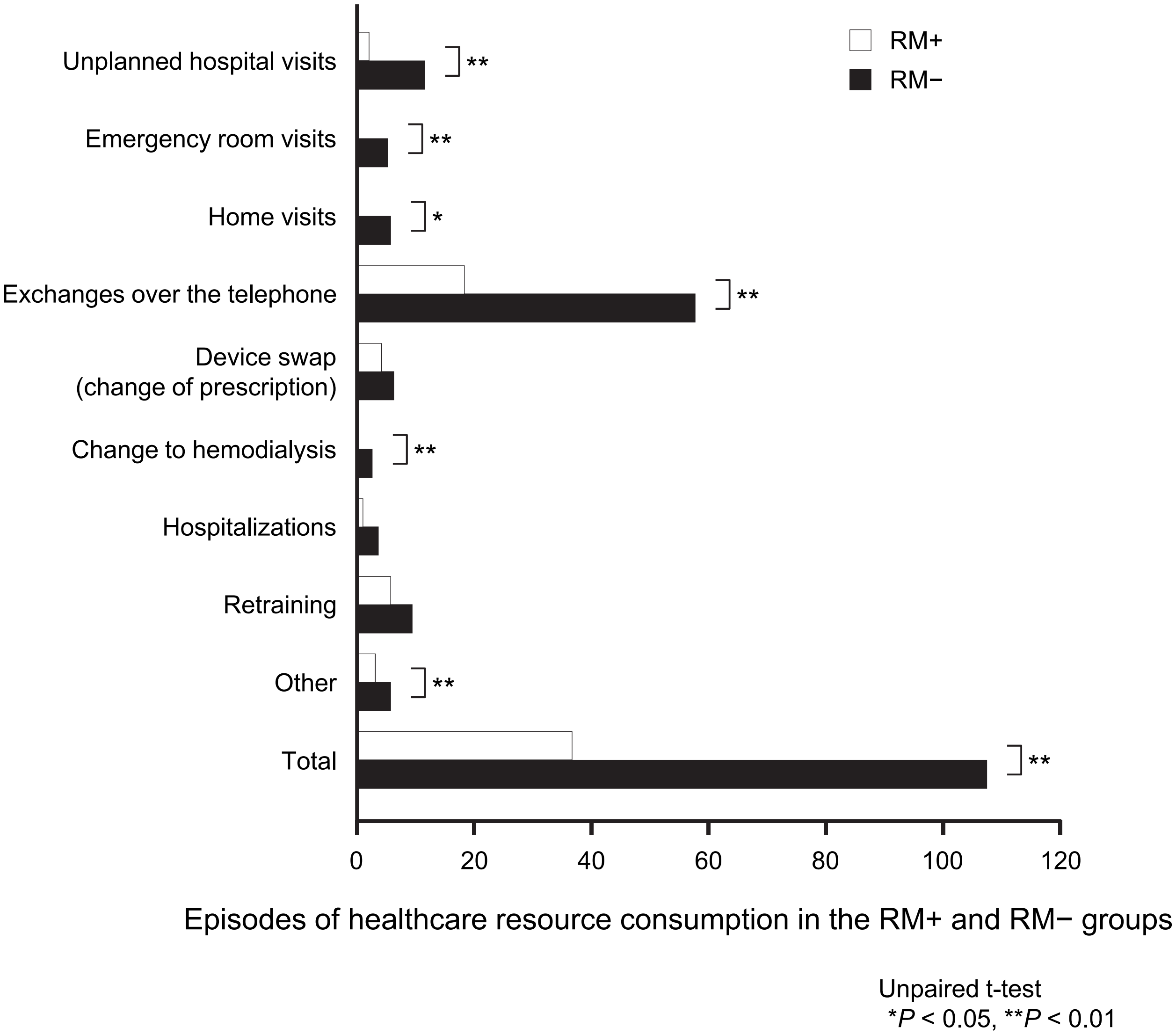

Supplementary Figure 1. Frequencies of healthcare resource consumption episodes in the RM+ and RM− groups. The total number of healthcare resource consumption episodes was significantly lower in the RM+ than in the RM− group (Student's t-test P = 0.002, Mann–Whitney U test P = 0.021).
RM, remote monitoring

RM+ group Patient 1. Scenario

1. Background

Patient	1	Gender	Female
Age	26		
Diagnosis	ESRD		
Comorbidities	Diabetes		
Current Therapy	APD for 3 years. All labs within normal range most of the time		
Living Arrangements			
The patient does not always record treatment data and skips clinic and laboratory appointments at times due to her busy schedule. When she reports to the clinic, she does not have treatment data accessible and has not been good about keeping a history of weight, blood pressure, device alarms, and overall therapy reporting. The clinic staff spends a lot of the patient's therapy time attempting to get data from her and informing her of the importance of data collection to improve or provide adequate therapy.			

2. Case Scenario

The patient has been recently (1 month) using a new APD device with remote monitoring capabilities.

Day 1: Patient arrives at clinic. Remote data treatment reviewed. Clinic staff note that everything is going well with the patient. No change to therapy.

Day 60: Healthcare audit of patient records occurs.

Patient 1. Evaluation

- Read the case scenario on the left page and select the immediate action to be taken from the list below. For options other than option 1, specify the number of additions or removals to be made for the resource.
- If your decision is to **“continue to monitor the situation and take no further action at this time”**, circle option 1 below. and proceed to evaluation of the next case.
- Circle “+” or “−” if you think the addition or removal of any healthcare resource is necessary in this scenario, respectively.
- Specify the number of additions or removals to be made for the resource.
- If you think any call is necessary for the patient for any of the purposes indicated in options 2-4 or 6-10 below, circle option 5 as well as the option you selected.

3. Evaluation

1. Continue to monitor the situation and take no further action at this time.
2. Unplanned hospital visits + / − visit(s)
3. Emergency room visits + / − visit(s)
4. Home visits + / − visit(s)
5. Exchanges over the telephone + / − times

   (including both calling from the patient and calling from the medical institution, as well as calling to the call center)
6. Device swap (including change of the prescription) + / − times
7. Change to hemodialysis + / − times
8. Hospitalizations + / − times
9. Retraining + / − times
10. Other, specify ____________ + / − times

Patient 2. Scenario

1. Background

Patient	2	Gender	Male
Age	20		
Diagnosis	ESRD (nephrotic syndrome)		
Comorbidities	None		
Current Therapy	APD, 2.5% glucose-containing PD solution		
Living Arrangements			
The patient was trained at a dialysis center in another city. No issues with training were observed at the initial clinic visit last month. The clinic staff reports the patient to be a very quick learner.<br><br>The patient attends a university and has good grades. He lives on campus in the dormitory without a roommate. The patient has a telephone in his room and a mobile (cell) phone.<br><br>The patient's parents live far away from the patient.			

2. Case Scenario

Day 1: The clinic staff proactively reviews remote treatment data and notices flags for this patient. It is noted that the patient's post-treatment blood pressures are low and that the patient's weight is below the target weight. The clinic staff calls the patient and determines that the patient's weight is low due to his soccer playing.

Patient 2. Evaluation

- Read the case scenario on the left page and select the immediate action to be taken from the list below. For options other than option 1, specify the number of additions or removals to be made for the resource.
- If your decision is to “continue to monitor the situation and take no further action at this time”, circle option 1 below and proceed to evaluation of the next case.
- Circle “+” or “−” if you think the addition or removal of any healthcare resource is necessary in this scenario, respectively.
- Specify the number of additions or removals to be made for the resource.
- If you think any call is necessary for the patient for any of the purposes indicated in options 2-4 or 6-10 below, circle option 5 as well as the option you selected.

3. Evaluation

1. Continue to monitor the situation and take no further action at this time.
2. Unplanned hospital visits + / − ____ visit(s)
3. Emergency room visits + / − ____ visit(s)
4. Home visits + / − ____ visit(s)
5. Exchanges over the telephone + / − ____ times

   (including both calling from the patient and calling from the medical institution, as well as calling to the call center)
6. Device swap (including change of the prescription) + / − ____ times
7. Change to hemodialysis + / − ____ times
8. Hospitalizations + / − ____ times
9. Retraining + / − ____ times
10. Other, specify ____________ + / − ____ times

Patient 3. Scenario

1. Background

Patient	3	Gender	Male
Age	65		
Diagnosis	ESRD		
Comorbidities	Diabetes, hypertension		
Current Therapy	About to start APD therapy. Minimal residual renal function and functionally anuric (50 mL/day). Peritoneal catheter placed 2 weeks ago. On 2 anti-hypertension medications.		
Living Arrangements			
The patient completed training today. He recently retired and lives alone.			

2. Case Scenario

Day 1: On his first night of therapy, the patient made 4 attempts to perform therapy without completing the treatment.

Day 2: As a part of standard care, the clinic leaves a phone message with the patient to follow up regarding first night of therapy. The patient calls back in the evening and leaves a message that he is okay. He is embarrassed to disclose his failures.

Day 3: The patient’s message is reviewed by the clinic staff. The clinic staff uses remote treatment data to help support discussion with the patient. Remote monitoring of subsequent treatments reveals the patient is still having problems.

Day 4: A telephone call is made to the patient to schedule retraining in person or via the phone.

Patient 3. Evaluation

- Read the case scenario on the left page and select the immediate action to be taken from the list below. For options other than option 1, specify the number of additions or removals to be made for the resource.
- If your decision is to “continue to monitor the situation and take no further action at this time”, circle option 1 below and proceed to evaluation of the next case.
- Circle “+” or “−” if you think the addition or removal of any healthcare resource is necessary in this scenario, respectively.
- Specify the number of additions or removals to be made for the resource.
- If you think any call is necessary for the patient for any of the purposes indicated in options 2-4 or 6-10 below, circle option 5 as well as the option you selected.

3. Evaluation

1. Continue to monitor the situation and take no further action at this time.
2. Unplanned hospital visits + / − ____ visit(s)
3. Emergency room visits + / − ____ visit(s)
4. Home visits + / − ____ visit(s)
5. Exchanges over the telephone + / − ____ times

   (including both calling from the patient and calling from the medical institution, as well as calling to the call center)
6. Device swap (including change of the prescription) + / − ____ times
7. Change to hemodialysis + / − ____ times
8. Hospitalizations + / − ____ times
9. Retraining + / − ____ times
10. Other, specify ____________ + / − ____ times

Patient 4. Scenario

1. Background

Patient	4	Gender	Male
Age	63		
Diagnosis	ESRD		
Comorbidities	Diabetic with cardiomyopathy; obese		
Current Therapy	APD, 1.5% glucose-containing PD solution overnight. 8,000 mL total therapy volume, 8 hours overnight with a last fill of 2000 mL Icodextrin. Stable on therapy. Last 2 clinic visits show patient tolerating therapy with occasional low volume drain alarms noted. Patient states he just repositions and alarms go away. No changes to current plan.		
Living Arrangements			
The patient was trained on APD 3 months ago with no problems. He is currently working as an engineer. He is married with 2 children both going to university and living at home.			

2. Case Scenario

Day 1-7: During APD therapy, the patient experiences multiple low volume drain alarms over the course of the last week and bypasses the alarms. The patient is annoyed with the alarms and calls technical support. The technical support staff reviews troubleshooting remedies with the patient. Technical support logs the event but does not contact the clinic.<br><br>Day 7: The clinic staff proactively reviews remote treatment data which reveal multiple low volume drain alarms.

Patient 4. Evaluation

- Read the case scenario on the left page and select the immediate action to be taken from the list below. For options other than option 1, specify the number of additions or removals to be made for the resource.
- If your decision is to "continue to monitor the situation and take no further action at this time", circle option 1 below and proceed to evaluation of the next case.
- Circle "+" or "−" if you think the addition or removal of any healthcare resource is necessary in this scenario, respectively.
- Specify the number of additions or removals to be made for the resource.
- If you think any call is necessary for the patient for any of the purposes indicated in options 2-4 or 6-10 below, circle option 5 as well as the option you selected.

3. Evaluation

1. Continue to monitor the situation and take no further action at this time.
2. Unplanned hospital visits + / −   visit(s)
3. Emergency room visits + / −   visit(s)
4. Home visits + / −   visit(s)
5. Exchanges over the telephone + / −   times

   (including both calling from the patient and calling from the medical institution, as well as calling to the call center)
6. Device swap (including change of the prescription) + / −   times
7. Change to hemodialysis + / −   times
8. Hospitalizations + / −   times
9. Retraining + / −   times
10. Other, specify ____________ + / −   times

Patient 5. Scenario

1. Background

Patient	5	Gender	Female
Age	34		
Diagnosis	ESRD		
Comorbidities	Lupus, hypertension, joint pain, sun sensitivity		
Current Therapy	On APD therapy for last 2 years. Patient has had good residual renal function. Recently sustained work injury and was given high-dose NSAIDs by the emergency department staff. Patient recently noted decrease in urine output.		
Living Arrangements			
The patient is working at a local manufacturing facility for the last 5 years.			

2. Case Scenario

Day 1: The patient visits the clinic for her regular monthly visit. The patient has labs collected and adequacy test performed during the visit. The patient has samples of effluent collected. The patient does not mention that she was recently prescribed high-dose NSAIDs.

Day 3: The remote treatment data display flags for weight and blood pressure for this patient. The laboratory results from the Day 1 visit are reviewed, and it is determined that renal function has decreased.

Patient 5. Evaluation

- Read the case scenario on the left page and select the immediate action to be taken from the list below. For options other than option 1, specify the number of additions or removals to be made for the resource.
- If your decision is to "continue to monitor the situation and take no further action at this time", circle option 1 below and proceed to evaluation of the next case.
- Circle "+" or "−" if you think the addition or removal of any healthcare resource is necessary in this scenario, respectively.
- Specify the number of additions or removals to be made for the resource.
- If you think any call is necessary for the patient for any of the purposes indicated in options 2-4 or 6-10 below, circle option 5 as well as the option you selected.

3. Evaluation

1. Continue to monitor the situation and take no further action at this time.
2. Unplanned hospital visits + / −   visit(s)
3. Emergency room visits + / −   visit(s)
4. Home visits + / −   visit(s)
5. Exchanges over the telephone + / −   times

   (including both calling from the patient and calling from the medical institution, as well as calling to the call center)
6. Device swap (including change of the prescription) + / −   times
7. Change to hemodialysis + / −   times
8. Hospitalizations + / −   times
9. Retraining + / −   times
10. Other, specify ____________ + / −   times

Patient 6. Scenario

1. Background

Patient	6	Gender	Female
Age	32		
Diagnosis	ESRD		
Comorbidities	Hypertension		
Current Therapy	APD therapy for 1 month; doing fine and has reached full fill volumes		
Living Arrangements			
The patient is a married school teacher with no children. She lives 3 hours away from the clinic.			

2. Case Scenario

Day 1: The patient visits the clinic for her regularly scheduled visit. During the visit, the clinic staff accidentally changed the patient's treatment data on the data card to a previous prescription with lower fill volumes. Adequacy test scheduled for next clinic visit. The patient performs therapy at home that evening and notices extra fluid left in the fluid bags at the end of treatment but does not question the settings.

Day 3: During review of remote treatment data, it is noted that the device is programmed incorrectly.

Patient 6. Evaluation

- Read the case scenario on the left page and select the immediate action to be taken from the list below. For options other than option 1, specify the number of additions or removals to be made for the resource.
- If your decision is to "continue to monitor the situation and take no further action at this time", circle option 1 below and proceed to evaluation of the next case.
- Circle "+" or "−" if you think the addition or removal of any healthcare resource is necessary in this scenario, respectively.
- Specify the number of additions or removals to be made for the resource.
- If you think any call is necessary for the patient for any of the purposes indicated in options 2-4 or 6-10 below, circle option 5 as well as the option you selected.

3. Evaluation

1. Continue to monitor the situation and take no further action at this time.
2. Unplanned hospital visits + / − visit(s)
3. Emergency room visits + / − visit(s)
4. Home visits + / − visit(s)
5. Exchanges over the telephone + / − times

   (including both calling from the patient and calling from the medical institution, as well as calling to the call center)
6. Device swap (including change of the prescription) + / − times
7. Change to hemodialysis + / − times
8. Hospitalizations + / − times
9. Retraining + / − times
10. Other, specify ____________ + / − times

Patient 7. Scenario

1. Background

Patient	7	Gender	Male
Age	76		
Diagnosis	Renal disease		
Comorbidities	Diabetic, severe visual impairment		
Current Therapy	APD		
Living Arrangements			
The patient and his wife live in their own home and use public transportation.			

2. Case Scenario

The patient is cared for at home by his elderly wife. She often makes mistakes during the treatment setup and needs to repeat the steps using new supplies each time. Because of the frequent setups, treatments are missed near the end of the month as she runs out of supplies. The wife is afraid that her husband will be placed in a nursing home due to her frequent mistakes with the treatment setup.

Day 1: The patient has a regular clinic visit. His wife answers all the questions and states that treatments are going well. Lab work is collected, and the sample the patient brought from home is sent out for analysis. Remote treatment data are reviewed during the clinic visit, and it is noted that the last few treatments are shortened or missed. After extensive discussion with the wife, it is determined that she is making mistakes with setups and using many additional supplies, causing this problem.

Patient 7. Evaluation

- Read the case scenario on the left page and select the immediate action to be taken from the list below. For options other than option 1, specify the number of additions or removals to be made for the resource.
- If your decision is to "continue to monitor the situation and take no further action at this time", circle option 1 below and proceed to evaluation of the next case.
- Circle "+" or "−" if you think the addition or removal of any healthcare resource is necessary in this scenario, respectively.
- Specify the number of additions or removals to be made for the resource.
- If you think any call is necessary for the patient for any of the purposes indicated in options 2-4 or 6-10 below, circle option 5 as well as the option you selected.

3. Evaluation

1. Continue to monitor the situation and take no further action at this time.
2. Unplanned hospital visits + / − visit(s)
3. Emergency room visits + / − visit(s)
4. Home visits + / − visit(s)
5. Exchanges over the telephone + / − times

   (including both calling from the patient and calling from the medical institution, as well as calling to the call center)
6. Device swap (including change of the prescription) + / − times
7. Change to hemodialysis + / − times
8. Hospitalizations + / − times
9. Retraining + / − times
10. Other, specify ____________ + / − times

Patient 8. Scenario

1. Background

Patient	8	Gender	Male
Age	45		
Diagnosis	ESRD secondary to diabetic nephropathy		
Comorbidities	Hypertension		
Current Therapy	Started dialysis 2 years ago; only treated with APD		
Living Arrangements			
The patient is self-employed and has been very busy at work. He lives alone at his house.			

2. Case Scenario

Day 5: Remote treatment data are reviewed, and missed/shortened treatments and elevated blood pressure and weight are noted. A telephone call is made to the patient to discuss remote treatment data and importance of adherence to treatment. Advise is given to the patient to change the glucose-containing PD solution from 1.5% to 2.5%

Patient 8. Evaluation

- Read the case scenario on the left page and select the immediate action to be taken from the list below. For options other than option 1, specify the number of additions or removals to be made for the resource.
- If your decision is to “continue to monitor the situation and take no further action at this time”, circle option 1 below and proceed to evaluation of the next case.
- Circle “+” or “−” if you think the addition or removal of any healthcare resource is necessary in this scenario, respectively.
- Specify the number of additions or removals to be made for the resource.
- If you think any call is necessary for the patient for any of the purposes indicated in options 2-4 or 6-10 below, circle option 5 as well as the option you selected.

3. Evaluation

1. Continue to monitor the situation and take no further action at this time.
2. Unplanned hospital visits + / − ____ visit(s)
3. Emergency room visits + / − ____ visit(s)
4. Home visits + / − ____ visit(s)
5. Exchanges over the telephone + / − ____ times

   (including both calling from the patient and calling from the medical institution, as well as calling to the call center)
6. Device swap (including change of the prescription) + / − ____ times
7. Change to hemodialysis + / − ____ times
8. Hospitalizations + / − ____ times
9. Retraining + / − ____ times
10. Other, specify ____________ + / − ____ times

Patient 9. Scenario

1. Background

Patient	9	Gender	Male
Age	44		
Diagnosis	ESRD		
Comorbidities	Diabetes, obesity with cardiomegaly; smoker (trying to quit)		
Current Therapy	APD treatment 8 months (effluent into the drain tank).		
Living Arrangements			
The patient is working full time at a factory on the assembly line. The patient is married with no children and lives in apartment complex close to work.			

2. Case Scenario

Day 3: Review of remote treatment data reveals multiple low volume drain alarms and treatment ending early. The patient is called, and after questioning him, a catheter problem is suspected. The patient is instructed to use a drain bag that evening and call the clinic the next day.<br><br>Day 4: The patient calls the clinic to state that he can see fibrin in the fluid.

Patient 9. Evaluation

- Read the case scenario on the left page and select the immediate action to be taken from the list below. For options other than option 1, specify the number of additions or removals to be made for the resource.
- If your decision is to "continue to monitor the situation and take no further action at this time", circle option 1 below and proceed to evaluation of the next case.
- Circle "+" or "−" if you think the addition or removal of any healthcare resource is necessary in this scenario, respectively.
- Specify the number of additions or removals to be made for the resource.
- If you think any call is necessary for the patient for any of the purposes indicated in options 2-4 or 6-10 below, circle option 5 as well as the option you selected.

3. Evaluation

1. Continue to monitor the situation and take no further action at this time.
2. Unplanned hospital visits + / − ____ visit(s)
3. Emergency room visits + / − ____ visit(s)
4. Home visits + / − ____ visit(s)
5. Exchanges over the telephone + / − ____ times

   (including both calling from the patient and calling from the medical institution, as well as calling to the call center)
6. Device swap (including change of the prescription) + / − ____ times
7. Change to hemodialysis + / − ____ times
8. Hospitalizations + / − ____ times
9. Retraining + / − ____ times
10. Other, specify ____________ + / − ____ times

Patient 10. Scenario

1. Background

Patient	10	Gender	Female
Age	87		
Diagnosis	ESRS (Nephrosclerosis)		
Comorbidities	Dementia, hypertension, blind		
Current Therapy	APD therapy		
Living Arrangements			
The patient is widowed and lives in a nursing home. APD therapy is performed by nursing staff.			

2. Case Scenario

Day 1: The nursing home staff phones the clinic to report the patient had multiple alarms during the night. The nursing home staff is unable to explain which alarms have occurred. The nursing home states they would like the patient to transfer to an in-center hemodialysis as they cannot manage the patient.

Day 3: Review of remote treatment data notes frequent low volume drain alarms and poor ultrafiltration. The clinic staff contacts the nursing home. After discussion with the nursing home staff, it is found that the patient's bed was lowered to avoid falls. The device height is changed to accommodate recent change in height of patient's bed.

Day 5: Review of remote treatment data notes continued frequent low volume drain alarms and poor ultrafiltration. The clinic staff contacts the nursing staff and after questioning determines the patient is constipated.

Patient 10. Evaluation

- Read the case scenario on the left page and select the immediate action to be taken from the list below. For options other than option 1, specify the number of additions or removals to be made for the resource.
- If your decision is to “continue to monitor the situation and take no further action at this time”, circle option 1 below and proceed to evaluation of the next case.
- Circle “+” or “−” if you think the addition or removal of any healthcare resource is necessary in this scenario, respectively.
- Specify the number of additions or removals to be made for the resource.
- If you think any call is necessary for the patient for any of the purposes indicated in options 2-4 or 6-10 below, circle option 5 as well as the option you selected.

3. Evaluation

1. Continue to monitor the situation and take no further action at this time.
2. Unplanned hospital visits + / − ____ visit(s)
3. Emergency room visits + / − ____ visit(s)
4. Home visits + / − ____ visit(s)
5. Exchanges over the telephone + / − ____ times

   (including both calling from the patient and calling from the medical institution, as well as calling to the call center)
6. Device swap (including change of the prescription) + / − ____ times
7. Change to hemodialysis + / − ____ times
8. Hospitalizations + / − ____ times
9. Retraining + / − ____ times
10. Other, specify ____________ + / − ____ times

Patient 11. Scenario

1. Background

Patient	11	Gender	Male
Age	21		
Diagnosis	Membranoproliferative glomerulonephritis type 2 (MPGN II)		
Comorbidities	Hypertension		
Current Therapy	APD therapy		
Living Arrangements			
The patient lives at home with 2 younger sisters, his mother, and grandmother in grandmother’s house. It is the summer time, and the patient is home from the university for this time He likes playing basketball and video games with his friends in the neighborhood.			

2. Case Scenario

Day 1: The patient is playing basketball during the hot summer days and is not drinking much fluid. He has experienced bad cramps and is dizzy but does not tell anyone.

Day 3: Review of the remote treatment data reveals missing weight and blood pressures. The clinic staff calls his family to investigate missed weights and blood pressures. The family states that they will enter the data.

Day 5: The patient complains of cramps and some dizziness to grandmother but states he is playing basketball and it is hard to keep up with the others because he is so small. The patient’s grandmother decides not to bother the patient’s mother when she gets home from work.

Meanwhile, review of remote treatment data shows that weight and blood pressure are down. The data also shows that the ultrafiltration has decreased. The clinic staff calls the patient and it is discovered that patient has been playing basketball every day has complained of dizziness and cramping.

Patient 11. Evaluation

- Read the case scenario on the left page and select the immediate action to be taken from the list below. For options other than option 1, specify the number of additions or removals to be made for the resource.
- If your decision is to “continue to monitor the situation and take no further action at this time”, circle option 1 below and proceed to evaluation of the next case.
- Circle “+” or “−” if you think the addition or removal of any healthcare resource is necessary in this scenario, respectively.
- Specify the number of additions or removals to be made for the resource.
- If you think any call is necessary for the patient for any of the purposes indicated in options 2-4 or 6-10 below, circle option 5 as well as the option you selected.

3. Evaluation

1. Continue to monitor the situation and take no further action at this time.
2. Unplanned hospital visits + / − visit(s)
3. Emergency room visits + / − visit(s)
4. Home visits + / − visit(s)
5. Exchanges over the telephone + / − times

   (including both calling from the patient and calling from the medical institution, as well as calling to the call center)
6. Device swap (including change of the prescription) + / − times
7. Change to hemodialysis + / − times
8. Hospitalizations + / − times
9. Retraining + / − times
10. Other, specify ____________ + / − times

Patient 12. Scenario

1. Background

Patient	12	Gender	Female
Age	21		
Diagnosis	Nephrotic syndrome/focal segmental glomerulosclerosis (FGCS)		
Comorbidities	Hypertension		
Current Therapy	APD for 5 years using a peritoneal dialysis cycler with data monitoring device (without remote monitoring). Lab indicates patient is becoming more uremic, resulting in a need to increase therapy.		
Living Arrangements			
The patient lives with her aunt and 2 younger sisters. Her parents are not a part of her life. Her aunt has legal custody and works full time and a part-time weekend job to earn additional money. The patient is in school with poor grades and a low attendance record. She is at risk of not graduating university.			

2. Case Scenario

The patient has been recently (1 month ago) using a new APD device that includes remote monitoring.

Day 1: Prior to the visit, the clinic staff reviews remote treatment data, and becomes aware that the patient is missing a number of treatments. The patient arrives at the clinic for the monthly visit. The patient is given counseling on the consequences of missed treatments and encouraged to adhere to the treatment.

Day 7: Remote treatment data are reviewed, and correct adherence confirmed.

Patient 12. Evaluation

- Read the case scenario on the left page and select the immediate action to be taken from the list below. For options other than option 1, specify the number of additions or removals to be made for the resource.
- If your decision is to “continue to monitor the situation and take no further action at this time”, circle option 1 below and proceed to evaluation of the next case.
- Circle “+” or “−” if you think the addition or removal of any healthcare resource is necessary in this scenario, respectively.
- Specify the number of additions or removals to be made for the resource.
- If you think any call is necessary for the patient for any of the purposes indicated in options 2-4 or 6-10 below, circle option 5 as well as the option you selected.

3. Evaluation

1. Continue to monitor the situation and take no further action at this time.
2. Unplanned hospital visits + / − ____ visit(s)
3. Emergency room visits + / − ____ visit(s)
4. Home visits + / − ____ visit(s)
5. Exchanges over the telephone + / − ____ times

   (including both calling from the patient and calling from the medical institution, as well as calling to the call center)
6. Device swap (including change of the prescription) + / − ____ times
7. Change to hemodialysis + / − ____ times
8. Hospitalizations + / − ____ times
9. Retraining + / − ____ times
10. Other, specify ____________ + / − ____ times

Please check for any missing entry.
This is the end.

RM– group Patient1 Scenario

1. Background

Patient	1	Gender	Female
Age	26		
Diagnosis	ESRD		
Comorbidities	Diabetes		
Current Therapy	APD for 3 years. All labs within normal range most of the time		
Living Arrangements			
The patient does not always record treatment data and skips clinic and laboratory appointments at times due to her busy schedule. When she reports to the clinic, she does not have treatment data accessible and has not been good about keeping a history of weight, blood pressure, device alarms, and overall therapy reporting. The clinic staff spends a lot of the patient's therapy time attempting to get data from her and informing her of the importance of data collection to improve or provide adequate therapy.			

2. Case Scenario

Day 1: The patient visits the clinic for her regularly scheduled visit. She states she forgot her daily records. The clinic staff questions the patient regarding her therapy, and she provides very little information. The patient is asked to mail her records to the clinic when she returns home. The patient does not mail the records since she never documented treatments.

Days 7-21: The clinic staff calls the patient every other day to remind her to send her records. The treatment plan is updated based on the patient's verbal reports.

Days 22-22: The patient still does not mail the records.

Day 31: The patient returns to the clinic for her regular scheduled visit and follow-up tests.

Day 33: The clinic calls the patient with updates and confirmation of changes.

Day 60: A review of clinic records from an audit/accreditation organization reveals this patient has missing treatment information. The facility is issued a warning. The clinic is instructed to prepare a care plan to address this issue.

Patient 1 Evaluation

3. Allocation of healthcare resources

In this scenario, the patient uses the following healthcare resources:

Healthcare resources used:

1. Eight (8) clinic calls (every other day between Days 7 to 21, Day 33)
2. One (1) detailed care plan in compliance with healthcare audit requirement

4. Evaluation

Evaluation 1: Do you agree that the use of the above healthcare resources constitutes the best clinical practice for the patient?

YES □ → Proceed to evaluation of the next case.

NO □ → Proceed to Evaluation 2 below.

Evaluation 2: If NO is selected above:

- Select the healthcare resource(s) from the list below that you think constitutes the best clinical practice for the patient within the treatment period of this scenario.
- Circle + or − if you think the addition or removal of any healthcare resource is necessary, respectively.
- Specify the number of additions or removals to be made for the resource.
- If the addition or removal of multiple resources is deemed necessary, indicate all that apply.
- Consider actions to be taken on days not indicated in the case scenario within the treatment period.

1. Unplanned hospital visits + / − ____ visit(s)
2. Emergency room visits + / − ____ visit(s)
3. Home visits + / − ____ visit(s)
4. Exchanges over the telephone + / − ____ times
   (including both calling from the patient and calling from the medical institution, as well as calling to the call center)
5. Device swap (including change of the prescription) + / − ____ times
6. Change to hemodialysis + / − ____ times
7. Hospitalizations + / − ____ times
8. Retraining + / − ____ times
9. Other, specify ____________ + / − ____ times

Patient 2. Scenario

1. Background

Patient	2	Gender	Male
Age	20		
Diagnosis	ESRD (nephrotic syndrome)		
Comorbidities	None		
Current Therapy	APD, 2.5% glucose-containing PD solution		
Living Arrangements			
The patient was trained at a dialysis center in another city. No issues with training were observed at the initial clinic visit last month. The clinic staff reports the patient to be a very quick learner.<br><br>The patient attends a university and has good grades. He lives on campus in the dormitory without a roommate. The patient has a telephone in his room and a mobile (cell) phone.<br><br>The patient's parents live far away from the patient.			

2. Case Scenario

Day 1: The patient calls the clinic and leaves the messages, "I got dizzy and almost fell in the lecture hall today," and "Overall I feel kind of bad". The message is reviewed by clinic staff. A return call is made, but the patient is not reached. The following message is left, "I got your message, give us a call; we are concerned and may need to make some changes."

Day 2: The patient feels fine and does not return the telephone call.

Day 3: The clinic staff attempts to contact the patient and leaves the following message, "It is very important that I talk to you. I am very concerned. Please call the on-call clinic staff and do not leave a message on the clinic phone".

Day 3 afternoon: The patient is taken to the local emergency room after he faints during soccer practice. The patient is administered intravenous fluids and instructed to follow up with the clinic the next day. The patient is discharged.

Day 4: The patient calls the clinic to discuss his emergency room visit but is unable to provide many details. The patient is asked to come in for an unplanned clinic visit today. The clinic staff requests that the emergency room fax the patient records to the clinic.

Day 5: The patient comes in for an unplanned visit, and his treatment is changed from 2.5% glucose-containing PD solution (to 1.5% glucose-containing PD solution due to hypovolemia and hypotension associated with increased sweating incurred while the patient plays soccer. The patient is instructed to call into the clinic daily for 1 week with blood pressures, weights, and ultrafiltration totals.

Days 6-12: Patients calls in daily with information. Patient is stable with current prescription changes.

Day 20: Clinic staff calls the patient but cannot talk with him directly. A message is left, but no return call from the patient. No information is provided about his dialysis treatment.

Patient 2 Evaluation

3. Allocation of healthcare resources

In this scenario, the patient uses the following healthcare resources:

Healthcare resources used:

1. One (1) emergency room visit
2. One (1) unplanned clinic visit
3. One (1) change of the prescription
4. Ten (10) clinic calls (Days 1, 3, 6-12)

4. Evaluation

Evaluation 1: Do you agree that the use of the above healthcare resources constitutes the best clinical practice for the patient?

YES □ → Proceed to evaluation of the next case.

NO □ → Proceed to Evaluation 2 below.

Evaluation 2: If NO is selected above:

・Select the healthcare resource(s) from the list below that you think constitutes the best clinical practice for the patient within the treatment period of this scenario.

・Circle + or − if you think the addition or removal of any healthcare resource is necessary, respectively.

・Specify the number of additions or removals to be made for the resource.

・If the addition or removal of multiple resources is deemed necessary, indicate all that apply.

・Consider actions to be taken on days not indicated in the case scenario within the treatment period.

1. Unplanned hospital visits +/− ____ visit(s)
2. Emergency room visits +/− ____ visit(s)
3. Home visits +/− ____ visit(s)
4. Exchanges over the telephone +/− ____ times

   (including both calling from the patient and calling from the medical institution, as well as calling to the call center)
5. Device swap (including change of the prescription) +/− ____ times
6. Change to hemodialysis +/− ____ times
7. Hospitalizations +/− ____ times
8. Retraining +/− ____ times
9. Other, specify ____________ +/− ____ times

Patient 3. Scenario

1. Background

Patient	3	Gender	Male
Age	65		
Diagnosis	ESRD		
Comorbidities	Diabetes, hypertension		
Current Therapy	About to start APD therapy. Minimal residual renal function and functionally anuric (50 mL/day). Peritoneal catheter placed 2 weeks ago. On 2 anti-hypertension medications.		
Living Arrangements			
The patient completed training today. He recently retired and lives alone.			

2. Case Scenario

Day 1: On his first night of therapy, the patient made 4 attempts to perform therapy without completing the treatment.

Day 2: As a part of usual practice, the clinic calls the patient by phone for feedback on first treatment night. Unable to reach the patient, a message is left asking the patient to call back for feedback on first night of therapy. The patient calls back in the evening and leaves a message that he is okay.

Day 3: The patient's message is reviewed by the clinic staff. The clinic staff tries to call the patient and leaves a message with a request for vital signs and therapy results.

Day 4-6: The patient is unable to resolve the issues with setup and stops trying. The clinic staff attempts to contact the patient repeatedly. The patient does not return the telephone calls.

Day 7: The patient is in the emergency room with severe fluid overload and pulmonary edema. There are discussions with the patient about further treatment. An acute hemodialysis catheter is placed and the patient is admitted to the hospital and started on hemodialysis.

Patient 3 Evaluation

3. Allocation of healthcare resources

In this scenario, the patient uses the following healthcare resources:

Healthcare resources used:

1. One (1) emergency room visit
2. Five (5) clinic calls (Day 2, 3, 4-6)
3. One (1) change to hemodialysis
4. One (1) hospitalization

4. Evaluation

Evaluation 1: Do you agree that the use of the above healthcare resources constitutes the best clinical practice for the patient?

YES □ → Proceed to evaluation of the next case.

NO □ → Proceed to Evaluation 2 below.

Evaluation 2: If NO is selected above:

・Select the healthcare resource(s) from the list below that you think constitutes the best clinical practice for the patient within the treatment period of this scenario.

・Circle + or − if you think the addition or removal of any healthcare resource is necessary, respectively.

・Specify the number of additions or removals to be made for the resource.

・If the addition or removal of multiple resources is deemed necessary, indicate all that apply.

・Consider actions to be taken on days not indicated in the case scenario within the treatment period.

1. Unplanned hospital visits + / − ____ visit(s)
2. Emergency room visits + / − ____ visit(s)
3. Home visits + / − ____ visit(s)
4. Exchanges over the telephone + / − ____ times

   (including both calling from the patient and calling from the medical institution, as well as calling to the call center)
5. Device swap (including change of the prescription) + / − ____ times
6. Change to hemodialysis + / − ____ times
7. Hospitalizations + / − ____ times
8. Retraining + / − ____ times
9. Other, specify ____________ + / − ____ times

Patient 4. Scenario

1. Background

Patient	4	Gender	Male
Age	63		
Diagnosis	ESRD		
Comorbidities	Diabetic with cardiomyopathy; obese		
Current Therapy	APD, 1.5% glucose-containing PD solution overnight. 8,000 mL total therapy volume, 8 hours overnight with a last fill of 2000 mL Icodextrin. Stable on therapy. Last 2 clinic visits show patient tolerating therapy with occasional low volume drain alarms noted. Patient states he just repositions and alarms go away. No changes to current plan.		
Living Arrangements			
The patient was trained on APD 3 months ago with no problems. He is currently working as an engineer. He is married with 2 children both going to university and living at home.			

2. Case Scenario

Day 1-7: During APD therapy, the patient experiences multiple low volume drain alarms over the course of the last week and bypasses the alarms. The patient is annoyed with the alarms and calls technical support. The technical support staff reviews troubleshooting remedies with the patient. Technical support logs the event but does not contact the clinic.

Days 7-9: The patient is still experiencing alarms and calls the clinic nurse (staff) to complain. He states he called technical support and completed all of the recommended tasks to address the alarms, so there must be something wrong with his machine. The clinic staff calls technical support to request the APD device be replaced the next day.

Day 10: The patient receives a new APD device.

Day 12: The patient calls the clinic a few days later and complains of abdominal pain. Effluent fluid is clear. The clinic staff reviews alarms and troubleshooting steps. The clinic staff documents the abdominal pain and clear fluid. The clinic staff inquires on the patient's frequency of bowel movements. The patient states that he is having hard stools for the last 4 days. The clinic staff prescribes a laxative to the patient.

Day 14: The patient calls to provide an update and states his abdominal pain is gone. The alarms have also stopped.

Patient 4 Evaluation

3. Allocation of healthcare resources

In this scenario, the patient uses the following healthcare resources:

Healthcare resources used:

1. Four (4) clinic calls (Days 7-9, 12, 14)
2. One (1) device swap
3. One (1) prescription added (laxative)

4. Evaluation

Evaluation 1: Do you agree that the use of the above healthcare resources constitutes the best clinical practice for the patient?

YES □ → Proceed to evaluation of the next case.

NO □ → Proceed to Evaluation 2 below.

Evaluation 2: If NO is selected above:

・Select the healthcare resource(s) from the list below that you think constitutes the best clinical practice for the patient within the treatment period of this scenario.

・Circle + or − if you think the addition or removal of any healthcare resource is necessary, respectively.

・Specify the number of additions or removals to be made for the resource.

・If the addition or removal of multiple resources is deemed necessary, indicate all that apply.

・Consider actions to be taken on days not indicated in the case scenario within the treatment period.

1. Unplanned hospital visits + / − ____ visit(s)
2. Emergency room visits + / − ____ visit(s)
3. Home visits + / − ____ visit(s)
4. Exchanges over the telephone + / − ____ times

   (including both calling from the patient and calling from the medical institution, as well as calling to the call center)
5. Device swap (including change of the prescription) + / − ____ times
6. Change to hemodialysis + / − ____ times
7. Hospitalizations + / − ____ times
8. Retraining + / − ____ times
9. Other, specify ____________ + / − ____ times

Patient 5. Scenario

1. Background

Patient	5	Gender	Female
Age	34		
Diagnosis	ESRD		
Comorbidities	Lupus, hypertension, joint pain, sun sensitivity		
Current Therapy	On APD therapy for last 2 years. Patient has had good residual renal function. Recently sustained work injury and was given high-dose NSAIDs by the emergency department staff. Patient recently noted decrease in urine output.		
Living Arrangements			
The patient is working at a local manufacturing facility for the last 5 years.			

2. Case Scenario

Day 1: The patient visits the clinic for her regular monthly visit. The patient has labs collected and adequacy test performed during the visit. The patient has samples of effluent collected. The patient does not mention that she was recently prescribed high-dose NSAIDs.

Day 6: The laboratory and adequacy results from the Day 1 visit are reviewed. It is determined that renal function has decreased and her dialysis prescription should be changed. The clinic staff tried to contact the patient and left a message that she needs to come to the clinic so that the changes to her therapy can be made.

Day 7-12: Multiple calls are made by the clinic staff without success; hence, no changes are made to her therapy.

Day 13: Finally, the clinic staff speaks with the patient who states she is unable to come to the clinic because of work and asks to make therapy changes herself.

The clinic staff instructs her over the phone on how to make the therapy changes to the APD device. The patient confirms she has followed the instructions and that changes were done.

Day 20: The patient becomes ill and goes to the emergency room with uremic symptoms. After her release, the patient calls the clinic to inform them of the emergency room visit and is asked to come into the clinic to be evaluated.

Day 21: The patient comes into the office for an unplanned clinic visit related to the emergency room visit. An investigation reveals that therapy was not changed correctly on device last week

Patient 5 Evaluation

3. Allocation of healthcare resources

In this scenario, the patient uses the following healthcare resources:

Healthcare resources used:

1. One (1) emergency room visit
2. One (1) unplanned clinic visit
3. Six (6) clinic calls (days 6, 7-12, 13, 20)

4. Evaluation

Evaluation 1: Do you agree that the use of the above healthcare resources constitutes the best clinical practice for the patient?

YES □ → Proceed to evaluation of the next case

NO □ → Proceed to Evaluation 2 below.

Evaluation 2: If NO is selected above:

- Select the healthcare resource(s) from the list below that you think constitutes the best clinical practice for the patient within the treatment period of this scenario.
- Circle + or − if you think the addition or removal of any healthcare resource is necessary, respectively.
- Specify the number of additions or removals to be made for the resource.
- If the addition or removal of multiple resources is deemed necessary, indicate all that apply.
- Consider actions to be taken on days not indicated in the case scenario within the treatment period.

1. Unplanned hospital visits + / − ____ visit(s)
2. Emergency room visits + / − ____ visit(s)
3. Home visits + / − ____ visit(s)
4. Exchanges over the telephone + / − ____ times

   (including both calling from the patient and calling from the medical institution, as well as calling to the call center)
5. Device swap (including change of the prescription) + / − ____ times
6. Change to hemodialysis + / − ____ times
7. Hospitalizations + / − ____ times
8. Retraining + / − ____ times
9. Other, specify ____________ + / − ____ times

Patient 6. Scenario

1. Background

Patient	6	Gender	Female
Age	32		
Diagnosis	ESRD		
Comorbidities	Hypertension		
Current Therapy	APD therapy for 1 month; doing fine and has reached full fill volumes		
Living Arrangements			
The patient is a married school teacher with no children. She lives 3 hours away from the clinic.			

2. Case Scenario

Day 1: The patient visits the clinic for her regular visit. During the visit, the clinic staff accidentally changed the patient's treatment data card to a previous prescription with lower fill volumes. Adequacy test scheduled for next clinic visit. The patient performs therapy at home that evening and notices extra fluid left in the fluid bags at the end of treatment but does not question the settings.

Day 31: The patient returns for her regularly scheduled visit. During the visit, the patient seems confused, and her family also reports a loss in appetite. The patient did not bring in her treatment data. The patient's weight and blood pressure are slightly elevated, but her family said this may be due to eating out before coming to the clinic. The patient reports no change in urine output. Lab work and adequacy samples are collected.

Day 33: Lab work and adequacy tests results are reviewed. The patient's creatinine and urea levels deteriorated from the previous month. The patient's treatment program is reviewed and it is noted that the wrong prescription was entered at the clinical visit 1 month ago. The clinic staff calls the patient's family and has them reprogram the cycle over the phone. The clinic staff instructs the patient to return in 2 weeks to repeat adequacy testing and lab work.

Day 57: The patient returns for an unplanned visit to repeat adequacy test and lab work.

Patient 6 Evaluation

3. Allocation of healthcare resources

In this scenario, the patient uses the following healthcare resources:

Healthcare resources used:

1. One (1) unplanned clinic visit
2. One (1) clinic call (day 33)
3. One (1) repeat lab work (urea, creatinine)
4. One (1) repeat adequacy test

4. Evaluation

Evaluation 1: Do you agree that the use of the above healthcare resources constitutes the best clinical practice for the patient?

YES □ → Proceed to evaluation of the next case

NO □ → Proceed to Evaluation 2 below.

Evaluation 2: If NO is selected above:

・Select the healthcare resource(s) from the list below that you think constitutes the best clinical practice for the patient within the treatment period of this scenario.

・Circle + or − if you think the addition or removal of any healthcare resource is necessary, respectively.

・Specify the number of additions or removals to be made for the resource.

・If the addition or removal of multiple resources is deemed necessary, indicate all that apply.

・Consider actions to be taken on days not indicated in the case scenario within the treatment period.

1. Unplanned hospital visits　+ / − ____ visit(s)
2. Emergency room visits　+ / − ____ visit(s)
3. Home visits　+ / − ____ visit(s)
4. Exchanges over the telephone　+ / − ____ times

   (including both calling from the patient and calling from the medical institution, as well as calling to the call center)
5. Device swap (including change of the prescription)　+ / − ____ times
6. Change to hemodialysis　+ / − ____ times
7. Hospitalizations　+ / − ____ times
8. Retraining　+ / − ____ times
9. Other, specify ____________　+ / − ____ times

Patient 7. Scenario

1. Background

Patient	7	Gender	Male
Age	76		
Diagnosis	Renal disease		
Comorbidities	Diabetic, severe visual impairment		
Current Therapy	APD		
Living Arrangements			
The patient and his wife live in their own home and use public transportation.			

2. Case Scenario

The patient is cared for at home by his elderly wife. She often makes mistakes during the treatment setup and needs to repeat the steps using new supplies each time. Because of the frequent setups, treatments are missed near the end of the month as she runs out of supplies. The wife is afraid that her husband will be placed in a nursing home due to her frequent mistakes with the treatment setup.

Day 1: The patient has a regular clinic visit. His wife answers all the questions and states that treatments are going well. Some of the treatment records are missing, and his wife states it is just because she gets involved in helping her husband and forgets to write everything down.

Lab work is collected, and the sample the patient brought from home is sent out for analysis. The clinic staff counsels the patient and his wife regarding the importance of recording all of the treatment information so he can get the best care. The wife agrees to try harder at remembering to record all information. No changes made to patients treatment regimen.

Day 31: The patient's wife calls clinic and states that her husband is not doing well and it is hard to get him up and going. The wife says he does not eat anything and seems to have difficulty breathing. The clinic staff instructs his wife to bring to the patient to the clinic, but she states she is unable due to his condition. The clinic staff tells the wife to go to the emergency room or call an ambulance. The wife calls the ambulance, and the patient is taken to the local emergency room. After an examination and lab work, the patient is admitted to the hospital with volume overload and uremia. After being admitted, it is determined that the wife is having difficulties with setups and that treatments have been missed.

Patient 7 Evaluation

3. Allocation of healthcare resources

In this scenario, the patient uses the following healthcare resources:

Healthcare resources used:

1. One (1) emergency room visit
2. One (1) hospitalization
3. One (1) counseling session with wife (patient re-training)

4. Evaluation

Evaluation 1: Do you agree that the use of the above healthcare resources constitutes the best clinical practice for the patient?

YES □ → Proceed to evaluation of the next case

NO □ → Proceed to Evaluation 2 below.

Evaluation 2: If NO is selected above:

・Select the healthcare resource(s) from the list below that you think constitutes the best clinical practice for the patient within the treatment period of this scenario.

・Circle + or − if you think the addition or removal of any healthcare resource is necessary, respectively.

・Specify the number of additions or removals to be made for the resource.

・If the addition or removal of multiple resources is deemed necessary, indicate all that apply.

・Consider actions to be taken on days not indicated in the case scenario within the treatment period.

1. Unplanned hospital visits + / − visit(s)
2. Emergency room visits + / − visit(s)
3. Home visits + / − visit(s)
4. Exchanges over the telephone + / − times

   (including both calling from the patient and calling from the medical institution, as well as calling to the call center)
5. Device swap (including change of the prescription) + / − times
6. Change to hemodialysis + / − times
7. Hospitalizations + / − times
8. Retraining + / − times
9. Other, specify ____________ + / − times

Patient 8. Scenario

1. Background

Patient	8	Gender	Male
Age	45		
Diagnosis	ESRD secondary to diabetic nephropathy		
Comorbidities	Hypertension		
Current Therapy	Started dialysis 2 years ago; only treated with APD		
Living Arrangements			
The patient is self-employed and has been very busy at work. He lives alone at his house.			

2. Case Scenario

Day 1: The patient calls the clinic to say his blood pressure is elevated. He states he is doing his treatment as prescribed and watching fluid intake but noted blood pressure has been increasing over the past 10 days to 150-155/90-100 (baseline 130/85). The patient reports no headache, shortness of breath, or puffiness. The patient says he has been taking his Losartan 50 mg daily. The patient's ultrafiltration is unchanged at 500-700 mL/treatment, and he reports his weight is stable at 76 Kg (dry weight 76 Kg). The patient is instructed to come in for an unplanned clinic visit.

Day 2: The patient comes in for an unplanned clinic visit and physical examination. The patient's blood pressure is 145/90, and his weight is 78 Kg. Lab work results are: BUN 90, K 5.2, HCO3 20, Phosphorous 8.0. The patient is questioned about the discrepancy between current lab results and previous adequacy test results (Kt/V = 1.8). The patient admits to the clinic staff that he has been stopping his treatments 1 hour early for the past 3 weeks to get to work early. The patient is instructed to change the glucose-containing PD solution from 1.5% to 2.5% until his blood pressure returns to the normal range.

Days 3-5: The patient has been calling the clinic over the last 3 days to report weight and blood pressure readings, which have improved. The patient is informed to return to using 1.5 % glucose-containing PD solution. The clinic staff also instructs the patient not to shorten his treatments. The patient is instructed to return to the clinic in 2 weeks for a previously unplanned visit to repeat lab work.

Day 22: The patient returns for an unplanned clinic visit. Lab work is obtained and event ends.

Patient 8 Evaluation

3. Allocation of healthcare resources

In this scenario, the patient uses the following healthcare resources:

Healthcare resources used:

1. One (2) unplanned clinic visit
2. Four (4) clinic calls (Day 1, 3-5)
3. One (1) repeat lab work
4. Two changes of the prescription

4. Evaluation

Evaluation 1: Do you agree that the use of the above healthcare resources constitutes the best clinical practice for the patient?

YES □ → Proceed to evaluation of the next case

NO □ → Proceed to Evaluation 2 below.

Evaluation 2: If NO is selected above:

・Select the healthcare resource(s) from the list below that you think constitutes the best clinical practice for the patient within the treatment period of this scenario.

・Circle + or − if you think the addition or removal of any healthcare resource is necessary, respectively.

・Specify the number of additions or removals to be made for the resource.

・If the addition or removal of multiple resources is deemed necessary, indicate all that apply.

・Consider actions to be taken on days not indicated in the case scenario within the treatment period.

1. Unplanned hospital visits + / − ____ visit(s)
2. Emergency room visits + / − ____ visit(s)
3. Home visits + / − ____ visit(s)
4. Exchanges over the telephone + / − ____ times

   (including both calling from the patient and calling from the medical institution, as well as calling to the call center)
5. Device swap (including change of the prescription) + / − ____ times
6. Change to hemodialysis + / − ____ times
7. Hospitalizations + / − ____ times
8. Retraining + / − ____ times
9. Other, specify ____________ + / − ____ times

Patient 9. Scenario

1. Background

Patient	9	Gender	Male
Age	44		
Diagnosis	ESRD		
Comorbidities	Diabetes, obesity with cardiomegaly; smoker (trying to quit)		
Current Therapy	APD treatment 8 months (effluent into the drain tank).		
Living Arrangements			
The patient is working full time at a factory on the assembly line. The patient is married with no children and lives in apartment complex close to work.			

2. Case Scenario

Day 0-7: While performing APD therapy at home, the patient has experienced multiple low volume drain alarms. The patient is annoyed with the alarms as it disturbs his sleep, and he frequently terminates treatment early.

Day 8: The patient calls technical support and states he is having multiple low volume drain alarms. He is very annoyed with these alarms and is not getting enough sleep. The technical support staff walks the patient through troubleshooting alarms.

Day 10: The patient calls the clinic to complain he is still having low volume drain alarms and has followed all of the troubleshooting suggestions provided by technical support. The patient states there is something wrong with the machine. The clinic staff has the device replaced.

Day 11: Device replaced.

Day 13: The patient calls the clinic to report shortness of breath, abdominal pain, and swelling of the hands and feet. The patient is advised to come in for unplanned clinic visit at the end of the day. The patient comes to clinic and explains he is having frequent low volume drain alarms. The patient does not provide any records. The patient does not report that he has been ending his treatments early due to lack of sleep. During the clinic visit, an exchange is done and fibrin is noted. It is suspected that the patient is having catheter problems due to fibrin.

Day 17: Patient calls and reports everything is fine.

Day 30: The patient does not appear although the day is a planned clinic visit date.

Patient 9 Evaluation

3. Allocation of healthcare resources

In this scenario, the patient uses the following healthcare resources:

Healthcare resources used:

1. One (1) unplanned clinic visit
2. One (1) device swap
3. Three(3) clinic calls (Days 10, 13, 17)

4. Evaluation

Evaluation 1: Do you agree that the use of the above healthcare resources constitutes the best clinical practice for the patient?

YES □ → Proceed to evaluation of the next case

NO □ → Proceed to Evaluation 2 below.

Evaluation 2: If NO is selected above:

・Select the healthcare resource(s) from the list below that you think constitutes the best clinical practice for the patient within the treatment period of this scenario.

・Circle + or − if you think the addition or removal of any healthcare resource is necessary, respectively.

・Specify the number of additions or removals to be made for the resource.

・If the addition or removal of multiple resources is deemed necessary, indicate all that apply.

・Consider actions to be taken on days not indicated in the case scenario within the treatment period.

1. Unplanned hospital visits +/− visit(s)
2. Emergency room visits +/− visit(s)
3. Home visits +/− visit(s)
4. Exchanges over the telephone +/− times

   (including both calling from the patient and calling from the medical institution, as well as calling to the call center)
5. Device swap (including change of the prescription) +/− times
6. Change to hemodialysis +/− times
7. Hospitalizations +/− times
8. Retraining +/− times
9. Other, specify ____________ +/− times

Patient 10. Scenario

1. Background

Patient	10	Gender	Female
Age	87		
Diagnosis	ESRS (Nephrosclerosis)		
Comorbidities	Dementia, hypertension, blind		
Current Therapy	APD therapy		
Living Arrangements			
The patient is widowed and lives in a nursing home. APD therapy is performed by nursing staff.			

2. Case Scenario

Day 1: The nursing home staff phones the clinic to report the patient had multiple alarms during the night. The nursing home staff is unable to explain which alarms have occurred. The nursing home states they would like the patient to transfer to an in-center hemodialysis as they cannot manage the patient.

Day 3: The dialysis nurse visits the nursing home to evaluate the alarms and patient catheter function. The nurse notes multiple low volume drain alarms. The dialysis nurse notes that the patient's bed had been lowered to avoid falls. The dialysis nurse instructs the nursing home staff that they need to adjust the height of device. The nursing home staff adjusts the height of the device as recommended. The nursing home staff was encouraged to contact the Peritoneal Dialysis unit if any further alarms occur.

Day 5: The nursing home staff is unable to note information on the alarms as they resolve before they can be noted. The nursing home staff calls the clinic and states again that the patient should be switched to hemodialysis. The dialysis nurse offers to provide training to the nursing home staff, but the staff refuses.

Day 19: As the patient is still experiencing low volume drain alarms, the nursing home facility manager contacts the patient's primary physician to discuss transfer to an in-center hemodialysis clinic. The primary physician contacts the patient's nephrologist. The nephrologist contacts the Peritoneal Dialysis unit staff and states that since PD cannot be managed, the patient will be changed to HD. The nursing care staff make transportation arrangements for treatment at an in-center hemodialysis clinic. The patient's therapy is changed to hemodialysis.

Patient 10 Evaluation

3. Allocation of healthcare resources

In this scenario, the patient uses the following healthcare resources:

Healthcare resources used:

1. One (1) change to hemodialysis
2. One (1) visit by PD staff to nursing home
3. Four (4) clinic calls (Days 1, 5, 19 (twice))

4. Evaluation

Evaluation 1: Do you agree that the use of the above healthcare resources constitutes the best clinical practice for the patient?

YES □ → Proceed to evaluation of the next case

NO □ → Proceed to Evaluation 2 below.

Evaluation 2: If NO is selected above:

・Select the healthcare resource(s) from the list below that you think constitutes the best clinical practice for the patient within the treatment period of this scenario.

・Circle + or − if you think the addition or removal of any healthcare resource is necessary, respectively.

・Specify the number of additions or removals to be made for the resource.

・If the addition or removal of multiple resources is deemed necessary, indicate all that apply.

・Consider actions to be taken on days not indicated in the case scenario within the treatment period.

1. Unplanned hospital visits + / − ____ visit(s)
2. Emergency room visits + / − ____ visit(s)
3. Home visits + / − ____ visit(s)
4. Exchanges over the telephone + / − ____ times

   (including both calling from the patient and calling from the medical institution, as well as calling to the call center)
5. Device swap (including change of the prescription) + / − ____ times
6. Change to hemodialysis + / − ____ times
7. Hospitalizations + / − ____ times
8. Retraining + / − ____ times
9. Other, specify ____________ + / − ____ times

Patient 11. Scenario

1. Background

Patient	11	Gender	Male
Age	21		
Diagnosis	Membranoproliferative glomerulonephritis type 2 (MPGN II)		
Comorbidities	Hypertension		
Current Therapy	APD therapy		
Living Arrangements			
The patient lives at home with 2 younger sisters, his mother, and grandmother in grandmother's house. It is the summer time, and the patient is home from the university for this time He likes playing basketball and video games with his friends in the neighborhood.			

2. Case Scenario

Day 1: The patient is playing basketball during the hot summer days and is not drinking much fluid. He has experienced bad cramps and is dizzy but does not tell anyone.

Day 3: The patient is not checking weight or blood pressure regularly. The patient complains of cramps and some dizziness to grandmother but states he is playing basketball and it is hard to keep up with the others because he is so small. The patient's grandmother decides not to bother the patient's mother when she gets home from work.

Day 5: The patient complains to his mother on the weekend that he is dizzy and feels weak. His mother calls the clinic after hours and states that the patient's blood pressure is much lower. His mother states that she is not aware of any therapy issues but that her son has not taken his vital signs over the last few nights. The on-call doctor reviews the data from last clinic visit in the computer and decides that one of the blood pressure medications should be decreased. The patient's mother is instructed to make the change.

Day 7: The patient faints while playing basketball. The patient is taken to the emergency room for dehydration. The mother is called at work to come to emergency room. The patient is discharged with instructions to follow up at the clinic tomorrow for an unplanned visit.

Day 8: The patient's mother takes the day off work to take her son to the clinic for an unplanned visit. The clinic staff reviews the emergency room report. The patient and his mother are instructed on the need to monitor weight and blood pressure and fluid balance during the summer months.

Days 8-15: The clinic staff calls the family daily to obtain vital signs, weight, and ultrafiltration

Patient 11 Evaluation

3. Allocation of healthcare resources

In this scenario, the patient uses the following healthcare resources:

Healthcare resources used:

1. One (1) emergency room visit
2. One (1) unplanned clinic visit
3. Eight (8) clinic calls (Days 5, 9-15)

4. Evaluation

Evaluation 1: Do you agree that the use of the above healthcare resources constitutes the best clinical practice for the patient?

YES □ → Proceed to evaluation of the next case

NO □ → Proceed to Evaluation 2 below.

Evaluation 2: If NO is selected above:

- Select the healthcare resource(s) from the list below that you think constitutes the best clinical practice for the patient within the treatment period of this scenario.
- Circle + or − if you think the addition or removal of any healthcare resource is necessary, respectively.
- Specify the number of additions or removals to be made for the resource.
- If the addition or removal of multiple resources is deemed necessary, indicate all that apply.
- Consider actions to be taken on days not indicated in the case scenario within the treatment period.

1. Unplanned hospital visits + / − visit(s)
2. Emergency room visits + / − visit(s)
3. Home visits + / − visit(s)
4. Exchanges over the telephone + / − times
   (including both calling from the patient and calling from the medical institution, as well as calling to the call center)
5. Device swap (including change of the prescription) + / − times
6. Change to hemodialysis + / − times
7. Hospitalizations + / − times
8. Retraining + / − times
9. Other, specify ____________ + / − times

Patient 12. Scenario

1. Background

Patient	12	Gender	Female
Age	21		
Diagnosis	Nephrotic syndrome/focal segmental glomerulosclerosis (FGCS)		
Comorbidities	Hypertension		
Current Therapy	APD for 5 years using a peritoneal dialysis cycler with data monitoring device (without remote monitoring). Lab indicates patient is becoming more uremic, resulting in a need to increase therapy.		
Living Arrangements			
The patient lives with her aunt and 2 younger sisters. Her parents are not a part of her life. Her aunt has legal custody and works full time and a part-time weekend job to earn additional money. The patient is in school with poor grades and a low attendance record. She is at risk of not graduating university.			

2. Case Scenario

Day 1: The patient arrives at the clinic for her monthly visit. Treatment information is given to the nurse. The clinic staff attempts to download treatment data without success as the data card is blank. The patient states she is not sure what has happened to the card, but she did put it into the machine. The clinic staff is unaware that the data card was erased to hide missed treatments. The clinic staff schedules a home visit.

Day 3: The patient calls the night before the home visit to cancel. The clinic staff calls to reschedule the home visit but does not receive a return call from the patient. The clinic staff calls the patient's aunt at her place of employment to schedule a home visit.

Day 4: The patient is surprised when the nurse shows up for the home visit. The patient states that everything is going great and shows the nurse her bedside setup. The nurse notices many boxes of supplies in the garage. The nurse asks the patients about the supplies and the aunt states that all supplies are kept in the house. The nurse determines by box count that many treatments have been missed over a long period of time. A plan is created and new data card is given to the patient.

Day 5-21: The nurse makes weekly calls to track progress. Review of the new data card on the next visit shows a decrease in missed treatments. The clinic staff is still unaware that the data card was erased in the past.

Patient 12 Evaluation

3. Allocation of healthcare resources

In this scenario, the patient uses the following healthcare resources:

Healthcare resources used:

1. One (1) home visit
2. Five (5) clinic calls (Day 3, Weekly 5-21)

4. Evaluation

Evaluation 1: Do you agree that the use of the above healthcare resources constitutes the best clinical practice for the patient?

YES □ → Proceed to evaluation of the next case

NO □ → Proceed to Evaluation 2 below.

Evaluation 2: If NO is selected above:

・Select the healthcare resource(s) from the list below that you think constitutes the best clinical practice for the patient within the treatment period of this scenario.

・Circle + or − if you think the addition or removal of any healthcare resource is necessary, respectively.

・Specify the number of additions or removals to be made for the resource.

・If the addition or removal of multiple resources is deemed necessary, indicate all that apply.

・Consider actions to be taken on days not indicated in the case scenario within the treatment period.

1. Unplanned hospital visits + / − visit(s)
2. Emergency room visits + / − visit(s)
3. Home visits + / − visit(s)
4. Exchanges over the telephone + / − times
   (including both calling from the patient and calling from the medical institution, as well as calling to the call center)
5. Device swap (including change of the prescription) + / − times
6. Change to hemodialysis + / − times
7. Hospitalizations + / − times
8. Retraining + / − times
9. Other, specify ____________ + / − times

Please check for any missing entry. This is the end.
